# Rallying and Amplifying Faculty Voice During Periods of Curriculum Reform

**DOI:** 10.1007/s40670-025-02500-x

**Published:** 2025-09-01

**Authors:** Adarsh Shidhaye, Kirby Allen, Shanna Williams, Kelly Quesnelle

**Affiliations:** Department of Biomedical Sciences, University of South Carolina School of Medicine Greenville, 607 Grove Road, Greenville, SC 29605 USA

**Keywords:** Medical education, Curriculum, Curricular reform, Curriculum revision, Faculty affairs

## Abstract

Faculty face numerous demands, leading to stress and burnout, which can be exacerbated during curricular reform. This study presents a new approach to curricular reform which takes advantage of people’s motivational propensities for learning and growth. Based on a self-determination framework, one medical science education department employed Kotter’s change model to implement a curricular reform through faculty working groups. High participation rates and measurable goal achievements were observed, highlighting the importance of motivation and structured support in driving curricular improvements and fostering a positive culture. This study presents an evidence-based model for curriculum reform tailored to medical science educational faculty.

## Background

According to data from the American Association of Medical Colleges, approximately 85–95% of allopathic medical schools in the United States are currently revising their curricula, planning to revise their curricula, or have recently completed such a change [1]. With this, a significant number of schools are implementing enhanced integration of basic and clinical science content and an increased use of technology and alternative self-directed learning modalities [1, 2]. As subject matter experts in their respective content domains, faculty play a crucial role in shaping the content and structure of medical education, which is essential for preparing successful future physicians. Planning and implementing successful curriculum reform necessitates a collective effort from faculty and staff, focusing on common, structured goals to prevent confusion, frustration, and other negative effects on the institution’s culture and employees.

Within an academic medical environment, however, faculty must balance additional administrative demands, patient support, research duties, and planning for personal career advancement along with their standard teaching responsibilities [3]. This can lead to higher stress and occupational burnout [4–6]. A sense of frustration due to time commitments and a lack of sense of community are the two most important predictors of faculty intent to leave institutions of higher education [4]. Further, significant relationships also exist between the stage of curriculum reform and levels of occupational burnout among faculty. Faculty with lower occupational burnout were more likely to be in the “action stage” of curriculum change, whereas faculty with higher levels of burnout were more likely to still be in the “attitude” or “intention” phase, suggesting that the anxiety of the daunting, inactionable task is more stressful than actually doing it [5].

With this in mind, the University of South Carolina School of Medicine Greenville (USC SOMG) employed a self-determination theoretical framework to curriculum reform, integrating Kotter’s eight steps for leading change model [7–9]. Self-determination theory (SDT) posits that humans have a natural inclination towards activity but are vulnerable to passivity if the appropriate conditions are not met. The conditions essential for enhanced performance and well-being should meet three basic psychological needs: autonomy, relatedness, and competence [7, 8]. In the context of curricular reform, these needs have been examined previously by Hansen and colleagues and manifest as the ability of faculty to make choices on their own regarding their work (i.e., autonomy), that the faculty member feels a part of the organization and can create relationships (i.e., relatedness), and the faculty member feels capable and successful in their role (i.e., competence) [10]. Conversely, lack of autonomy in top-down curricular reform approaches can lead to disengagement, and similar structures can also lead uninvolved expert faculty to feel isolated or experience a lack of relatedness. Social environments during curricular reform that disregard the psychological needs described by SDT risk undermining faculty competence, leading to disengagement and intent to leave the institution. Social environments supportive of these needs, however, promote greater internalization (‘taking in’ of a value) and integration (transformation of that value into one’s sense of self [7].

Kotter’s eight steps for leading change model provides insight into the interrelatedness of variables inside a social environment which help to facilitate positive motivation and change [9]. Developed in 1995 by John Kotter, a Professor at the Harvard Business School, these guiding steps involve creating a sense of urgency to motivate stakeholders, building a guiding coalition to lead the effort, and developing a clear vision and strategy. Communicating the vision widely ensures understanding and buy-in, while empowering broad-based action removes obstacles and enables others to act on the vision. Generating short-term wins builds momentum, and consolidating gains drives further change. Finally, anchoring new approaches in the organizational culture ensures sustainability [9].

## Activity

**Fig. 1:**
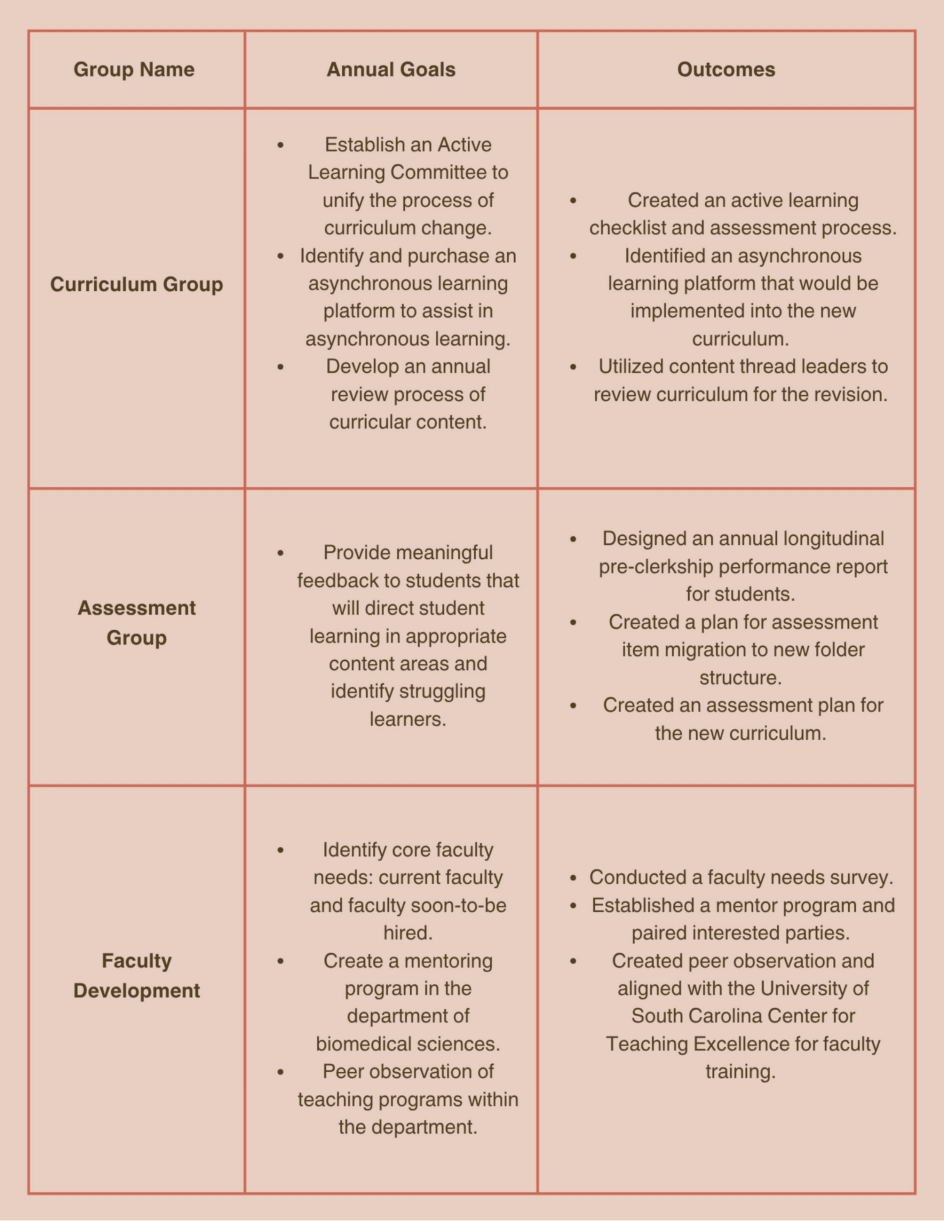
Working groups with associated annual goals and outcomes

Considering the daily competing interests vying for faculty attention, departmental leadership at USC SOMG designed a multi-tiered approach that promoted programmatic interests to increase faculty motivation in curricular reform. During the developmental and implantation stages, faculty voices were amplified through the formation of specialized working groups, surveys, and incentives. Through collaboration with approximately 20 departmental faculty, specific focus areas for the upcoming academic year were created, leading to the formation of three primary areas of development: curricular modalities, assessment of medical student’s progress, and faculty professional development. Each of these areas was tied to a working group of core faculty members with specific measurable goals designed by each group (Fig. 1). This approach was aimed at promoting autonomy and collaboration across the biomedical sciences faculty and ensuring that their input was intentionally solicited in a structured way to drive the pending pre-clerkship phase curricular reform and strategic plans for the school. Working groups ran through the first full iteration of the pre-clerkship phase of the curriculum revision, and faculty were free to choose any work group after an initial presentation to the department about the goals of each working group.

## Results

When given the option to join a working group, 60% of faculty chose to participate and received an additional $1000 in professional development funds for completing working group goals during the first year of the program. Groups met monthly and reported their findings at faculty meetings where all processes and products were vetted and approved by the larger faculty prior to implementation. The goals of each working group were achieved by the start of the next academic year (Fig. 1). Following a successful first year of working group activity with 60% of the department participating, 84% of faculty at our institution elected to join a working group for the second academic year, with all participants involved in initial working groups electing to rejoin. Importantly, a committee of faculty leaders in the department elected to remove incentives for joining groups since the institution was able to raise faculty development funds for all departmental faculty by $1000, regardless of working group participation.

## Discussion

Implementing large-scale curricular reform is not a quick or easy endeavor, but gathering and honoring faculty voices is an essential part of the process. The formation and products of active working groups based on faculty-determined academic priorities at our institution, and following Kotter’s change model, provided the scaffolding to allow individuals to construct the social conditions required to optimize extrinsically motivated behavior (i.e., autonomy, competence, and relatedness). In contrast to intrinsic motivation, which refers to activity engagement due to inherent satisfaction in the activity itself, most activities performed in adulthood are extrinsically motivated by social pressures. Extrinsic motivation involves behavior driven by reasons other than their inherent satisfaction (i.e., external stimuli or outcomes) [7, 8]. Self-Determination theory specified four types of extrinsic motivation (external, introjected, identified, and integrated regulation) which exist along a continuum ranging from highly controlled to autonomous. Integrated regulation of extrinsic motivation is the most autonomous form and represents an individual’s willingness to act due to conscious endorsement of the value of an activity. Environments which provide *autonomy support* for individuals promote satisfaction in both autonomy and relatedness. When combined with *structure,* these environments also promote competence gratification [7]. Our working group approach to curricular reform created such an environment for these basic psychological needs to be met. We used Kotter’s steps for leading change [9] to create conditions that supported the psychological needs of autonomy, competence, and relatedness. For example, building a guiding coalition supports the SDT component of relatedness by fostering collaboration and relationship-building among faculty, helping them feel connected to a shared purpose. Forming a strategic vision and initiatives promotes both autonomy and competence by involving faculty in shaping the direction and goals of the reform, allowing them to exercise choice and align their contributions with their professional strengths.

**Fig. 2:**
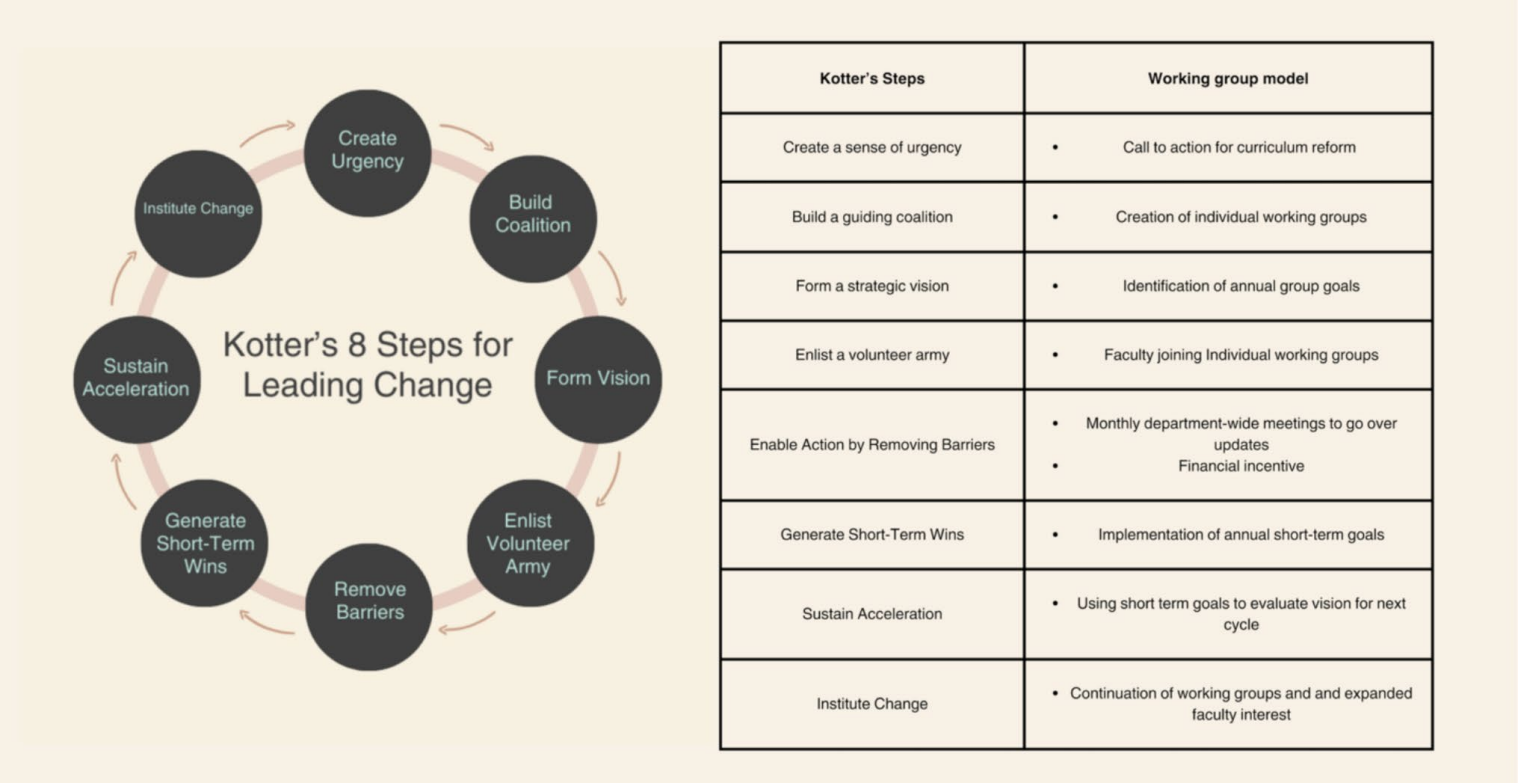
Kotter’s 8 steps of accelerating change integrated into the working group model. *Note: “Enlist a volunteer army” is drawn directly from Kotter’s framework for leading change, and it serves as a metaphor for inclusive and voluntary engagement in the change process*

Kotter’s model provided a structured approach to implement and execute change within a defined period (Fig. 2). The announcement of curricular reform for the 2023–2024 academic year created a sense of urgency, which prompted the construction of a guiding coalition in the formation of working groups that faculty were invited to join. The provision of choice allowed faculty to self-select to engage in a working group. This sense of choice allowed for greater ownership of activities, a sense of belonging and growth potential in their established competencies for those selecting similar areas of interest. Strategic vision was made manifest through the identification of annual, measurable goals for each working group. Potential barriers to involvement were minimized via regular communication of actions and financial incentives. Implementation of annual, measurable goals allowed for reportable short-term wins. This helped to maintain momentum in the working groups and fostered analytical evaluation of the vision for the next cycle, relative to clarification and/or revision. Finally, institutionalizing change was evident in the continuation of the works groups and annual re-evaluation of their goals, as well as expanded interest in faculty involvement during the second cycle even with the removal of financial incentives. While initial participation was incentivized with professional development funds, the autonomy-supportive structure of the intervention allowed for internalization of motivation, consistent with SDT, as evidenced by increased voluntary engagement after incentives were removed.

We acknowledge the limitation of this work that it was a program conducted with one group of faculty at one medical school. Other factors such as peer influence, perceived institutional support, or changes in workload may have contributed to expanded faculty involvement. Further, while this work does address many operational elements of a faculty engagement model, other strategic dimensions like conflict resolution and decision-making authority are not addressed here. However, we believe our outcomes are highly encouraging and we believe this work warrants future study at other programs undergoing curricular revision, providing an opportunity to study this process with more robust qualitative or mixed-method methodologies.

## Conclusion

The implementation of Kotter's eight steps for leading change to address the psychological needs of faculty according to self-determination theory has proven to be an effective strategy for curricular reform at the USC SOMG. By addressing the basic psychological needs of autonomy, relatedness, and competence, the institution created a supportive environment that fostered faculty engagement and motivation. The formation of working groups allowed faculty to actively participate in the reform process, enhancing their sense of ownership and collaboration. The success of the initial working groups, evidenced by high participation rates and the achievement of measurable goals, demonstrates the potential for sustained and meaningful change. As the institution continues to refine and expand its approach, the emphasis on faculty voices and structured support will remain crucial in driving ongoing curricular improvements and promoting a positive academic environment. This study addresses a significant gap in the literature, as there is a lack of evidence-based models for change in curriculum reform within medical education. By describing a model rooted in change theory but specifically tailored to medical science educational faculty, this research provides a valuable framework for other institutions seeking to implement similar reforms.

## Data Availability

Not applicable.
